# Exercise-induced modulation of myokine irisin on muscle-bone unit in the rat model of post-traumatic osteoarthritis

**DOI:** 10.1186/s13018-024-04532-2

**Published:** 2024-01-09

**Authors:** Xingru Shang, Xiaoxia Hao, Wenjie Hou, Jiawei Liu, Ruimin Chi, Xiaofeng Deng, Chunran Pan, Tao Xu

**Affiliations:** Department of Rehabilitation, Tongji Hospital, Tongji Medical College, Huazhong University of Science and Technology, 1095#, Jie-Fang Avenue, Qiaokou District, Wuhan, 430030 Hubei China

**Keywords:** Post-traumatic osteoarthritis, Treadmill training, Muscle–bone unit, Irisin

## Abstract

**Background and aim:**

Post-traumatic osteoarthritis (PTOA) is a subtype of osteoarthritis (OA). Exercise may produce and release the myokine irisin through muscle fiber contraction. However, the effect of exercise-promoted irisin production on the internal interactions of the muscle–bone unit in PTOA studies remains unclear.

**Methods:**

Eighteen 8-week-old Sprague–Dawley (SD) rats were randomly divided into three groups: Sham/sedentary (Sham/Sed), PTOA/sedentary (PTOA/Sed), and PTOA/treadmill-walking (PTOA/TW). The PTOA model was established by transection of anterior cruciate ligament (ACLT) and destabilization of medial meniscus (DMM). After 4 weeks of modeling, the PTOA/TW group underwent treadmill exercise (15 m/min, 30 min/d, 5 d/ week, 8 weeks), and the other two groups were free to move in the cage. Evaluation and correlation analysis of muscle, cartilage, subchondral bone and serological indexes were performed after euthanasia.

**Results:**

Eight weeks of treadmill exercise effectively alleviated the trauma-induced OA phenotype, thereby maintaining cartilage and subchondral bone integrity in PTOA, and reducing quadriceps atrophy and myofibril degradation. Exercise reversed the down-regulated expression of peroxisome proliferator-activated receptor-gamma coactivator-1α (PGC-1α) and fibronectin type III structural domain protein 5 (FNDC5) in muscle tissue of PTOA rats, and increased the blood irisin level, and the irisin level was positively correlated with the expression of PGC-1α and FNDC5. In addition, correlation analysis showed that irisin metabolism level was strongly negatively correlated with Osteoarthritis Research Society International (OARSI) and subchondral bone loss, indicating that irisin may be involved in cartilage biology and PTOA-related changes in cartilage and subchondral bone. Moreover, the metabolic level of irisin was strongly negatively correlated with muscle fiber cross-sectional area (CSA), Atrogin-1 and muscle ring-finger protein-1(MuRF-1) expression, suggesting that irisin may alleviate muscle atrophy through autocrine action.

**Conclusion:**

Treadmill exercise can alleviate the atrophy and degeneration of muscle fibers in PTOA rats, reduce the degradation of muscle fibrin, promote the expression of serum irisin, and alleviate the degeneration of articular cartilage and subchondral bone loss in PTOA rats. These results indicate that treadmill exercise can affect the process of PTOA by promoting the expression of myokine irisin in rat muscle–bone unit.

## Introduction

Osteoarthritis (OA) represents a degenerative ailment primarily characterized by alterations within the articular cartilage, subchondral bone, periarticular musculature, and synovial tissue of joints. This frequently results in symptoms like stiffness, inflammation, pain, restricted range of motion, and compromised joint function. Remarkably prevalent in the elderly, this condition stands as a significant contributor to diminished quality of life due to its debilitating effects [1, 2].

Post-traumatic osteoarthritis (PTOA) is considered to be a subtype of OA, accounting for roughly 12% of all instances of symptomatic OA. Among dominant risk factors may contribute to PTOA, incidence of anterior cruciate ligament (ACL) injury that progresses to PTOA is as high as 87%. In addition to ACL injury, many other associated alterations including the damage of articular cartilage, meniscus and subchondral bone and ligament laxity as well as atrophy or arthrogenic muscle inhibition may be concurrent during initial trauma and subsequent instability [3, 4].

Numerous evidences have revealed that a tight functional and developmental relationship exists between muscle and bone mass, which was identified as muscle–bone unit. Thus, in the complex muscle–bone cross-talk, both tissues are complementary and necessary for locomotion and individual activities [5]. Exercise, as a nonsurgical and non-pharmacological strategy, which is considered to be safe and effective in OA treatment, mainly regarding the alleviation of pain and improvement of physical function [6]. However, the mechanism of exercise in the prevention and treatment of OA between muscle and bone driven by mechanical stress are still not clearly illuminated. Current studies have indicate that exercise-induced skeletal muscle contraction may trigger the synthesis and secretion of myokines, thus connecting muscles with other human organs such as gut, bone and brain, and also acting on themselves through myokines [7]. The identification of myokines and their pivotal roles in regulating metabolic processes may provide new ideas to interpret the protective effects of exercise in treating OA [8].

Irisin is a newly identified myokine. Under the intervention of exercise, irisin is mainly secreted by skeletal muscle in large amount and released in the blood stream, participating in the resistance to inflammation and aging, which is a possible explanation for the protective and preventive mechanism of exercise on bones and joints [8]. Its precursor is fibronectin type III domain protein 5 (FNDC5), and the domain is fibronectin III. As a transmembrane protein, its C-terminal fragment exists in the interior of the cell membrane, and its N-terminal fragment is exposed to the outside of the cell. After cleavage, the N-terminal fragment located outside the cell is hydrolyzed to produce a protein, named irisin.[9].

As a secretory protein, irisin is an N-glycosylated protein hormone consists of 112 amino acids, which travel out systemic circulation and act on the target organs to play physiological parts [10]. A study conducted by He et al. exhibited that irisin exhibited a remarkable role to salvage bone volume (BV) fraction and trabecular number (Tb.N) in mice OA model induced by transection of the anterior cruciate ligament (ACLT). This intervention concurrently enhanced bone mineral density (BMD) by counteracting osteoblast apoptosis [11]. Furthermore, scientific literature has documented the anti-OA impact of irisin in chondrocytes [12]. These collective findings have paved the way for our hypothesis, which posits that irisin might indeed serve as a pivotal driving force in OA therapy. Studies have shown that peroxisome proliferator-activated receptor-γ (PPAR-γ) and its coactivator-1-α (PGC-1α) is increased in skeletal muscle after exercise, and the production of its downstream protein FNDC5 is increased to form the cleavage product irisin [13]. A recent study has demonstrated that moderate-intensity treadmill exercise protects against chondrocyte inflammation and pyroptosis through increasing the levels of irisin in OA rat models. However, the mechanisms by which exercise-induced irisin promotes the interaction between muscle and bone to achieve therapeutic effect on PTOA remains unclear [14].

In this study, our hypothesis centers on the potential of exercise training to ameliorate the advancement of PTOA by triggering the activation of the PGC-1α/FNDC5/irisin signaling pathway, thereby fostering the intrinsic interplay within the muscle-bone unit. To investigate this hypothesis, we subjected a PTOA rat model to a regimen of moderate exercise training. Subsequently, we scrutinized the impact on muscle, cartilage, subchondral bone, and serological factors, while assessing their interrelationships. This comprehensive approach aims to unravel the intricate links between the regulatory influence of treadmill exercise on irisin serum expression, the dynamics of the muscle-bone unit, and its consequential impact on the progression of PTOA.

## Methods

### Animals

The experimental animals were Eighteen 8-week-old Sprague–Dawley(SD) rats underwent adaptive training on a treadmill for 1 week at an intensity of 10 m/min, 10 min/d, and 5 d/ week rats with a weight of 250 ± 10 g, which provided by the Experimental Animal Centre of Tongji Medical College, Huazhong University of Science and Technology (Wuhan). All animals were kept in SPF-grade environment for long-term experiments. The Experimental Animal Ethics Committee authorized the experimental protocol. (approval number: TJH-202007010). Every two animals were housed in a plastic cage with irradiated sawdust bedding in a steady temperature (22 ± 2 °C) and humidity (60 ± 5%) room for 12 h on a cycle between day and night. The animals are given unrestricted access to food, drink, and movement within their enclosures. All experimental animal behaviors strictly adhered to the International Association for the Study of Pain's appropriate animal protection and usage laws.

### Exercise protocol and experimental design

**Fig. 1:**
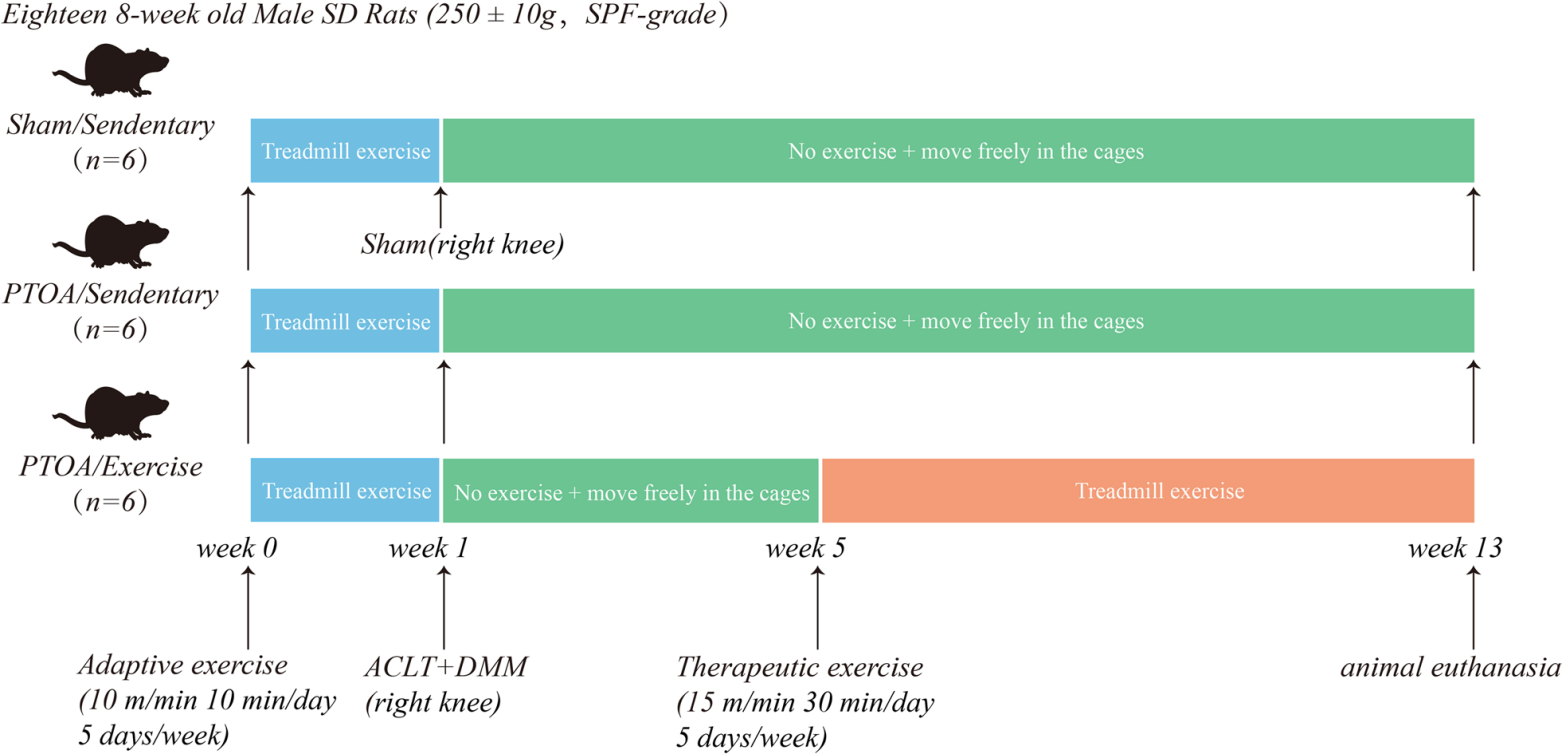
Eighteen SD rats underwent adaptive training on a treadmill for 1 week at an intensity of 10 m/min, 10 min/d, and 5 d/week. After one week of training, rats were randomly divided into three groups: Sham/sedentary (Sham/Sed), PTOA/sedentary (PTOA/Sed), and PTOA/treadmill-walking (PTOA/TW). The PTOA model was established by transection of anterior cruciate ligament (ACLT) and destabilization of medial meniscus (DMM). While the rats in the sham group were sutured after opening the right knee capsule and no extra surgery was undertaken. The PTOA/TW group performed 8 weeks of treadmill exercise at an intensity of 15 m/min, 30 min/d, 5 d/week. All SD rats were euthanized within 48 h of the last treadmill exercise, blood supernatant was collected after cardiac puncture, and tissue samples were collected and weighed

In Fig. 1, the study's protocols are displayed. Eighteen SD rats underwent adaptive training on a treadmill for 1 week at an intensity of 10 m/min, 10 min/d, and 5 d/week (Zhenghua Biological Instrument Equipment, China). After one week of training, the rats were randomly assigned to one of two groups: sham (*n* = 6) or PTOA (*n* = 12). Isoflurane anesthesia was used during all surgical procedures. The capsule of the right knee joint was opened in the PTOA rats, was subjected to the ACLT and the destabilization of the medial meniscus (DMM), and the joint capsule and skin were sutured layer by layer after disinfection of the joint cavity. While the rats in the sham group were sutured after opening the right knee capsule and no extra surgery was undertaken. The PTOA group was randomly divided into two groups four weeks after surgery: PTOA/sedentary (PTOA/Sed) group (*n* = 6) and PTOA/treadmill-walking (PTOA/TW) group (*n* = 6), while the Sham/sedentary (Sham/Sed) group (*n* = 6) received all of the sham animals. We chose exercise protocol based on earlier study that exercise training at 15 m/min, 30 min/day, 5 d/week, 8 weeks was effective in maintaining cartilage and subchondral bone integrity, and could reduce cartilage degeneration, systemic inflammation and mechanical pain [15, 16].For eight weeks, the rats in the Sham/Sed and PTOA/Sed groups were free to move around in their cages. These rats were allowed to rest on the treadmill for 30 min each day, but no running platform exercise was conducted. All SD rats were euthanized within 48 h of the last treadmill exercise. Blood supernatant was collected after cardiac puncture, and tissue samples were collected and weighed.

### Micro-CT analysis of subchondral bone

The intact knee joint of the animal was removed and fixed with 4% paraformaldehyde solution for 24–48 h. Then the knee joints of each group were scanned by micro-CT (micro-CT 50 Scanco Medical, Switzerland) at 100 kV, 98μA, and 10.5 μm resolution. The three-dimensional reconstruction of tibial plateau was performed using the three-dimensional image processing system of micro-CT software. The analysis software of the system was used to quantitatively analyze the subchondral bone area at the same position of the tibial plateau reconstruction image of SD rats. The analysis indicators included: bone volume/tissue volume fraction (BV/TV), trabecular number (Tb.N), trabecular separation (Tb.Sp) and trabecular thickness (Tb.Th).

### Histological analysis of articular cartilage

Following a micro-CT scan of the knee, it was decalcified for 4 weeks using a 10% ethylenediaminetetraacetic acid (EDTA) solution. Following dehydration with a graded ethanol solution, the specimen is embedded in paraffin. Slices were cut in the sagittal plane of the joint, with 4 µm of each section collected for histological examination. The tissue and morphological characteristics of the slices were studied under a microscope after they were stained with hematoxylin–eosin (HE) and safranin O (SafO)/fast green. The Osteoarthritis Research Society International (OARSI) scoring system was employed to evaluate the degree of damage at the histopathological level of cartilage in SD rats (0–6 points), which has a high sensitivity and reproducibility for the histopathological manifestations of cartilage in PTOA rats. The medial tibial plateau (MTP) was selected for scoring, and scores were summed and reported, with higher scores suggesting higher histopathological grading of OA.

### Immunohistochemical analysis of articular cartilage

Slices of the joint were prepared in the sagittal plane, with each slice measuring 4 µm in thickness for immunohistochemical analysis. Tissue expression of matrix metalloproteinases-13 (MMP-13, 1:100; proteintech, 18,165–1-AP) and collagen type II alpha 1 (COL2A1, 1:100; proteintech, 28,459–1-AP) was assessed through immunohistochemistry. The computer image analysis technique (Image-Pro Plus) was employed to analyze the percentage of positively stained cells in all the cartilage slices.

### Muscle mass/mass ratio

Within 48 h of the last exercise, the SD rats were placed on a weighing scale and after quieting, the net weight was measured and recorded. The quadriceps muscle was removed after execution and the excess tissue was removed, and the wet weight was weighed and recorded. The mass ratio of quadriceps muscle/body weight was calculated.

### Histological analysis of muscle

The separated intact quadriceps muscle was paraformaldehyde fixed, ethanol dehydrated and then paraffin embedded. Tissue slices and HE staining were performed on the muscle belly of the quadriceps muscle to assess the muscle fiber cross-sectional area (CSA) size and convert it into digital images. A computer-based image analysis technique (Image-Pro Plus) was used to analyze muscle hypertrophy by measuring the CSA of the abdominal fibers of the quadriceps muscle by measuring the area of 100 fibers located at 5 different sites.

### Immunohistochemical analysis of muscle

The effect of treadmill exercise on quadriceps muscle atrophy and the PGC-1α/FNDC5/irisin axis under OA was assessed using immunohistochemical staining. Tissue slices were performed in the abdominal region of the quadriceps muscle and positive tissue expression of Atrogin-1 (1:100; proteintech, 67,172–1-IG), muscle ring-finger protein-1 (MuRF-1, 1:100; proteintech, 55,456–1-AP), PGC-1α (1:100; proteintech, 66,369 1-IG) and FNDC5 (1:100; proteintech, 23,995–1-AP) were detected by immunohistochemistry. All sections were analyzed for the percentage of positive tissue staining using computer-based image analysis technology (Image-Pro Plus).

### The expression of mRNA levels of muscle

RNA was extracted from muscle using a total RNA extraction kit (Omega Biotek, R6834-01). It was then reverse transcribed into cDNA using Hifair®III 1st Strand cDNA Synthesis SuperMix (Yeasen, 11141ES60). cDNA sequences were amplified using SYBR Green Master Mix (Yeasen, 11203ES03). The pri-mers sequences were as follows: *FNDC5* (F) 5′-TAACCGTCAGGCACCTCAAGG-3′, (R) 5′-CGCAGCATCCTCACATCCTTC-3′. *MurF-1* (F) 5′-CTGCTGGTGGAGAACATCATCG-3′, (R) 5′-TTCTCGTCTTCGTGTTCCTTGC-3′. *Atrogin-1* (F) 5′-GGTCCAGAGAGTCGGCAAGTC-3′, (R) 5′-GGCAGGTCGGTGATCGTGAG-3′. *β-actin* (F) 5′-CTGTGTTGTCCCTGTATGCCTCTG-3′, (R) 5′-GGAACCGCTCATTGCCGATAGTG-3′. Each cDNA sample was repeated at least three times.

### Serum biochemical analysis

The animals were anesthetized with isoflurane before euthanasia, after which the blood supernatant was collected by cardiac puncture and temporarily stored at − 80 °C for subsequent analysis. Enzyme-linked immunosorbent assay (ELISA) was used to measure serum irisin concentration (RA21204; Bio-Swamp, China).

### Statistical analysis

GraphPad Prism 8.0 was used for statistical analysis of the data in this experimental study. The study employed one-way analysis of variance (ANOVA) to facilitate comparisons among multiple groups, while independent samples *t*-test were employed to compare the two groups, and the results were reported as mean ± standard error. When *p*-value < 0.05, the difference was considered statistically significant. Nonparametric Spearman correlation analysis was used to analyze the correlation between the two factors. When *p* value < 0.05, the difference was considered statistically significant.

## Results

### Exercise attenuates PTOA-relevant phenotypes of cartilage and subchondral bone

**Fig. 2:**
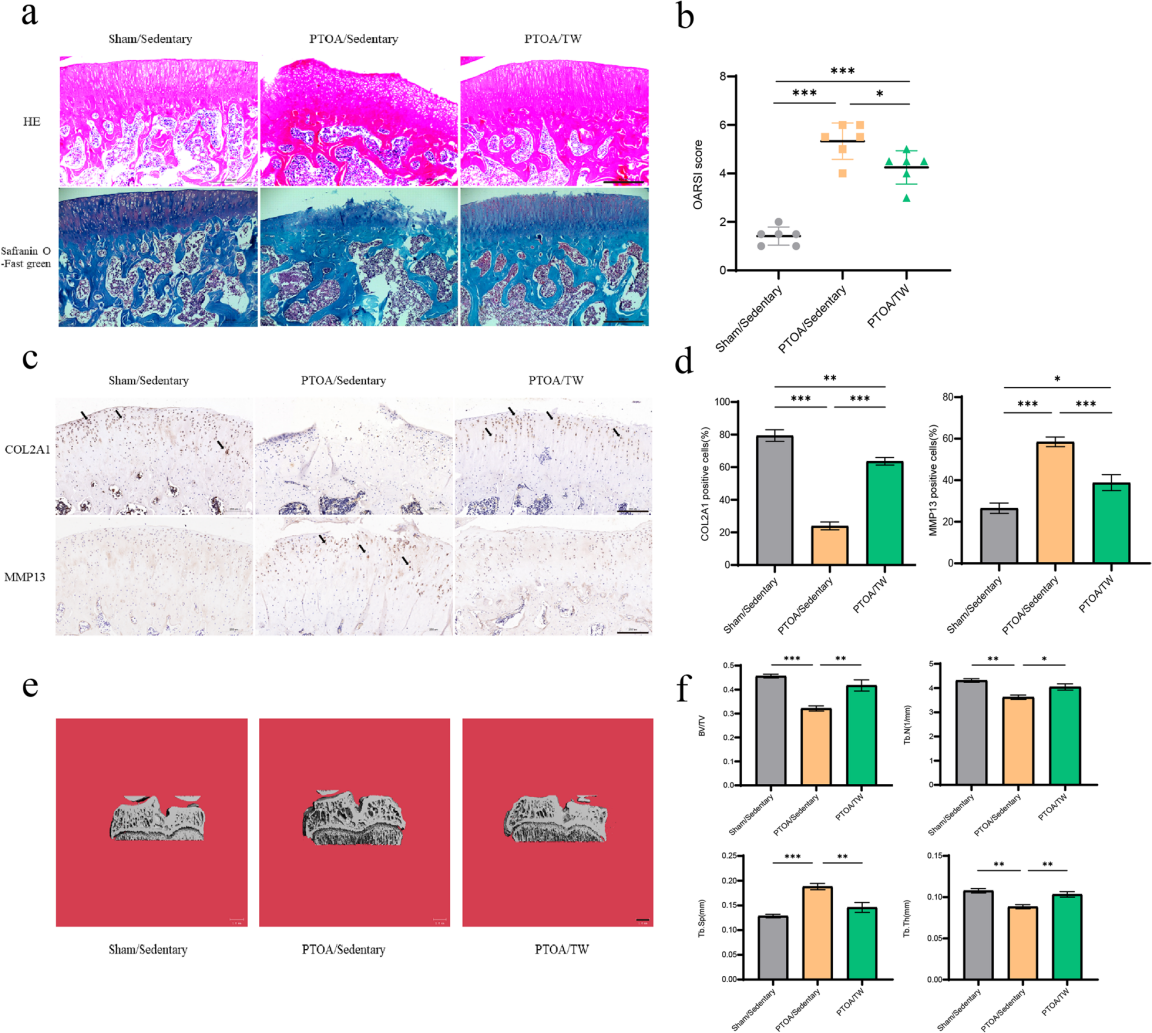
Exercise attenuates PTOA-relevant phenotypes of cartilage and subchondral bone. **a** The representative images of hematoxylin–eosin (HE) and safranin O (SafO)/fast green staining (scale bar = 400 μm, black) in a sagittal plane. **b** Osteoarthritis Research Society International (OARSI) score of the Sham/Sed, PTOA/Sed, and PTOA/TW groups. **c** Immunohistochemical staining of COL2A1 and MMP13 positive cells was performed in tibial plateau sections of each group (scale bar = 200 μm, black). **d** Percentage of COL2A1 and MMP13 positive cells in each group. **e** Three-dimensional micro-CT reconstruction of tibial coronal plane of rats in each group (scale bar = 1 mm, white). **f** Statistical analysis of tibial trabecular parameters: volume/tissue volume fraction (BV/TV), trabecular number bone (Tb.N), trabecular separation (Tb.Sp) and trabecular thickness (Tb.Th). The mean and standard error are used to represent the data, *n* = 6. * < 0.05, ** < 0.01, *** < 0.001

At 12 weeks post-surgery, the histological phenotypes of the cartilage and subchondral bone in the PTOA/Sed group exhibited evident articular cartilage degeneration and subchondral bone damage. As demonstrated in Fig. 2a, degeneration of cartilage in the PTOA/Sed group was defined by cartilage clefts, disordered chondrocyte sequences, and loss of cartilage matrix. These histological changes were further confirmed by OARSI scoring (Fig. 2b). Immunohistochemical staining of tibial sections from the PTOA/Sed group demonstrated a higher proportion of MMP13-positive cells in the cartilage matrix compared to the Sham/Sed group, while the proportion of COL2A1-positive cells was lower (Fig. 2c and d). These findings collectively suggest an accelerated cartilage degeneration in the PTOA model. Micro-CT results revealed significant bone loss and remodeling in the subchondral bone of the PTOA/Sed group (Fig. 2e and f). In the PTOA animal model, these changes imply a decreased cartilage and subchondral bone integrity. However, 8 weeks of treadmill exercise effectively mitigated trauma-induced OA phenotypes, thereby maintaining cartilage health and subchondral bone structure in PTOA. In comparison to the PTOA/Sed group, the PTOA/TW group exhibited reduced cartilage degeneration, decreased OARSI scores, a lower proportion of MMP13-positive cells, and an increased proportion of COL2A1-positive cells in the cartilage matrix after 8 weeks of postoperative treadmill exercise. Additionally, a sustained reduction in subchondral bone reaction characterized by decreased bone loss was displayed in the exercised animals (Fig. 2).

### Exercise increases quadriceps muscle volume and improves muscle atrophy

**Fig. 3:**
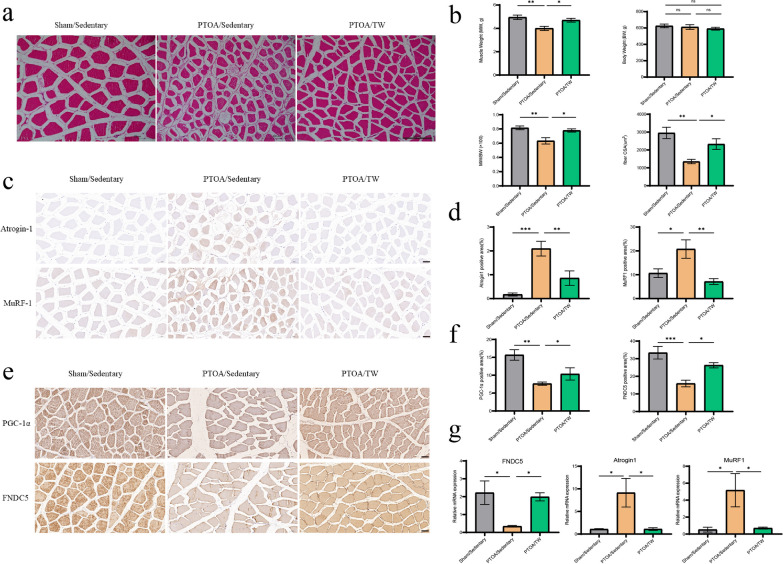
Exercise increases quadriceps muscle volume and improves muscle atrophy. **a** Histological sections of quadriceps femoris in each group were stained with HE (scale bar = 200 μm, black). **b** Body weight and quadriceps femoris mass/mass ratio in each group. **c** Immunohistochemical staining of Atrogin-1 and MuRF-1 was performed in quadriceps femoris muscle sections of each group (scale bar = 50 μm, black). **d** Percentage of Atrogin-1 and MuRF-1 positive area in each group. **e** Immunohistochemical staining of PGC-1α and FNDC5 was performed in quadriceps femoris muscle sections of each group (scale bar = 50 μm, black). **f** Percentage of PGC-1α and FNDC5 positive area in each group. **g** The mRNA levels of FNDC5、Atrogin-1 and MuRF-1 in each group by qRT-PCR. The mean and standard error are used to represent the data, *n* = 6. * < 0.05, ** < 0.01, *** < 0.001. ns not significant

At 12 weeks post-surgery, histological staining of muscle tissues using HE staining in the Sham/Sed group revealed regular and well-arranged muscle fibers. In contrast, the PTOA/Sed group displayed shorter muscle fiber diameters, indicative of atrophic changes (Fig. 3a). The three groups of rats' body weights did not significantly differ from one another. The PTOA/Sed group exhibited a trend of lower quadriceps muscle mass/mass ratio compared to the Sham/Sed group (Fig. 3b). Immunohistochemical staining was performed to detect the expression of muscle atrophy-related proteins, Atrogin-1 and MuRF-1, and the mRNA expression levels were detected by qRT-PCR. The proportion of positive staining and mRNA levels of Atrogin-1 and MuRF-1 tended to increase in the PTOA/Sed group compared with the Sham/Sed group (Fig. 3c, d and g). These changes suggest severe quadriceps muscle fiber atrophy in the PTOA animal model. However, 8 weeks of treadmill exercise effectively mitigated quadriceps muscle atrophy and reduced muscle fiber protein degradation. In comparison to the PTOA/Sed group, the PTOA/TW group significantly improved the quadriceps muscle mass/mass ratio, increased CSA scores, and decreased the proportion of muscle fiber which related to the production of Atrogin-1 and MuRF-1(Fig. 3).

### Exercise increase serum levels of irisin and PGC-1α and FNDC5 expression in muscle

**Fig. 4:**
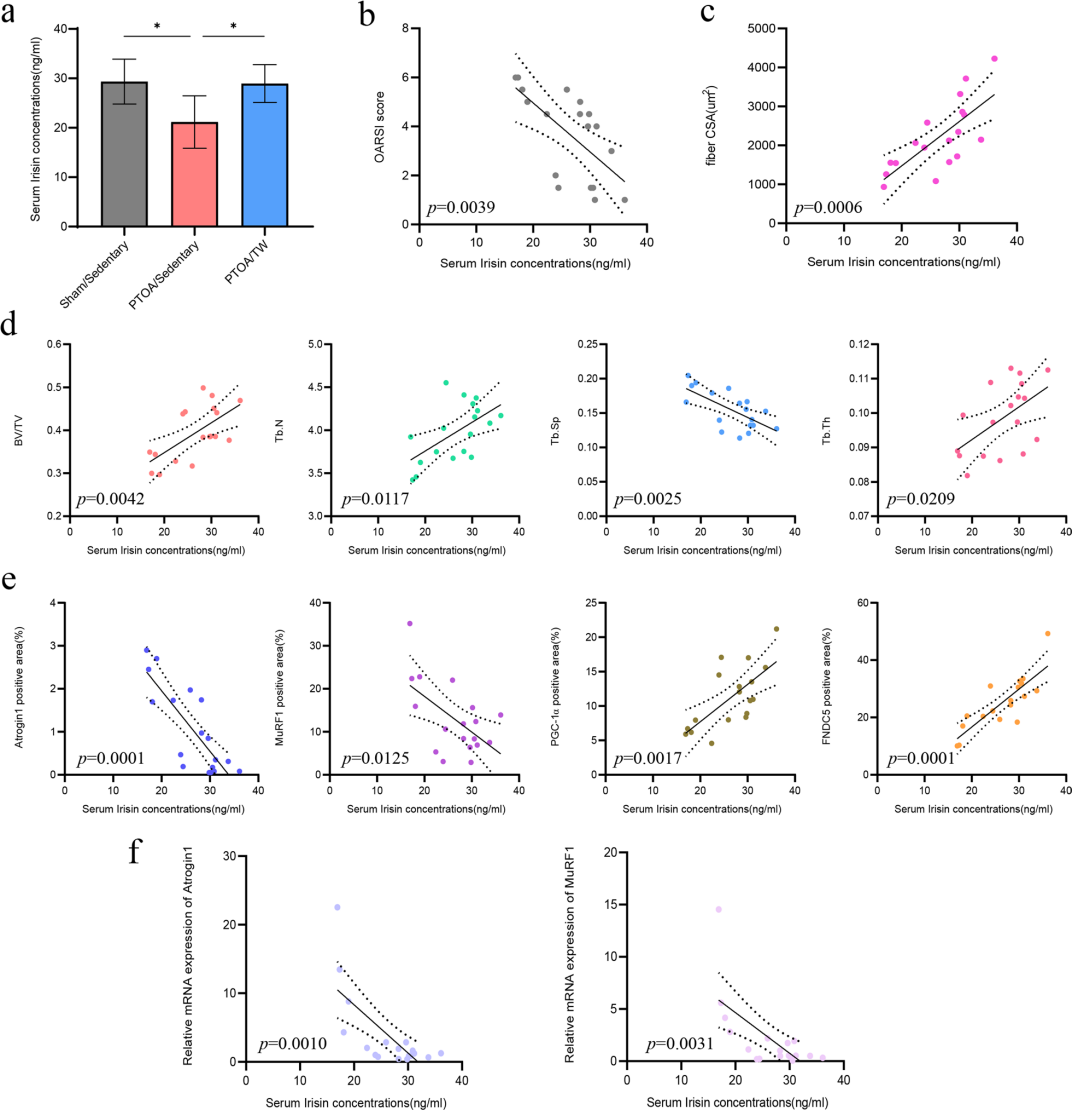
Exercise-induced changes in irisin concentration are related to the health of the cartilage, the structure of the subchondral bone, and the extent of muscle atrophy. **a** Enzyme-linked immunosorbent assay(ELISA) analysis of serum Irisin concentrations in each group. *n* = 6. * < 0.05. **b** Correlation analysis between Osteoarthritis Research Society International (OARSI) score and serum Irisin concentration. **c** Correlation analysis between muscle fiber cross-sectional area (CSA) and serum Irisin concentration. **d** Correlation analysis between subchondral bone micro-CT parameters and serum Irisin concentration. **e** Correlation analysis of percentage of Atrogin-1, MuRF-1, PGC-1α and FNDC5 positive area to serum Irisin concentration. **f** Correlation analysis between mRNA expression of Atrogin-1 as well as MuRF-1 and serum Irisin concentration. *n* = 18

We evaluated the expression of PGC-1α and FNDC5 in quadriceps muscle by immunohistochemical analysis. At 12 weeks post-surgery, the expression of PGC-1α and FNDC5 was downregulated in the PTOA/Sed group. In parallel, we performed qRT-PCR experiments and observed a significant decrease in FNDC5 mRNA expression levels. Surprisingly, 8 weeks of treadmill exercise reversed the downregulation of PGC-1α and FNDC5 expression levels in the muscle tissue of PTOA rats (Fig. 3e, f and g). Additionally, the impact of exercise on serum levels of irisin was investigated by ELISA. ELISA results revealed decreased serum irisin levels in the PTOA/Sed group. However, 8 weeks of treadmill exercise significantly increased blood levels of metabolically active irisin (Fig. 4a). Moreover, the metabolic status of irisin was negatively correlated with the expression of PGC-1α and FNDC5 positive tissues (Fig. 4e). These outcomes collectively hint at the prospect that treadmill exercise could potentially enhance the expression of PGC-1α and FNDC5 within the quadriceps femoris, leading to an augmented concentration of metabolized irisin in the muscle. Together with these findings, we speculated that the PGC-1α/FNDC5 signaling axis may be involved in the mechanisms of exercise-induced increased serum irisin concentration.

### Exercise activate PGC-1α/FNDC5-irisin signaling axis to promote muscle–bone cross-talk

Taking into account the impacts of treadmill exercise on cartilage, subchondral bone, and quadriceps muscle, we used correlation analysis to further investigate the connection between these impacts. As shown in Fig. 4, we established correlations encompassing various factors, including joint structural evaluation index (OARSI score and micro-CT data), muscle atrophy-related parameters (CSA and expression levels of Atrogin-1 and MuRF-1), and irisin levels. Intriguingly, a compelling negative correlation emerged between irisin serum levels and both OARSI scores and subchondral bone loss (Fig. 4b and d). This striking observation implies that irisin might actively participate in cartilage biology while concurrently exerting influence over PTOA-associated changes in the subchondral bone. Furthermore, the metabolic status of irisin exhibited a robustly negative correlation with CSA measurements, as well as with the expression of Atrogin-1 and MuRF-1 (Fig. 4c, e and f). This compelling association suggests that irisin might potentially counteract muscle atrophy through autocrine pathway.

These results suggested the underlying mechanism of PTOA alleviation by moderately intensive exercise, which significantly increased serum irisin concentration and promoted the internal interaction of the muscle–bone unit of the knee joint.

## Discussion

**Fig. 5:**
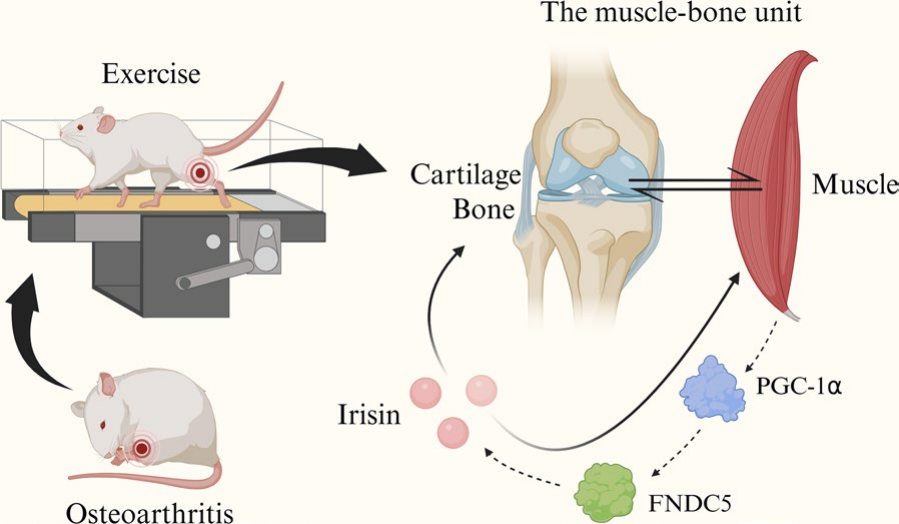
Cartilage, bone, and muscle are intricately interlinked, constituting what is known as the muscle-bone unit. The muscles are key producers of the myokine irisin. Moderate-intensity exercise can promote the release of irisin by activating the PGC-1α/FNDC5/irisin signal axis in muscle, maintain the health of the cartilage and the structure of the subchondral bone, and improve muscle atrophy. Exercise can improve OA by promoting muscle–bone cross-talk of the knee joint (created with biorender.com)

PTOA is a type of OA. OA due to trauma is also common in young adults. For example, in the USA alone, 6–8% of active youth experience menisci injuries each year, and this number is increasing in older age groups. In fact, according to a 2020 epidemiological study of young adults, approximately 13% of patients with meniscus injury develop OA 18 years after injury. The 8-week-old rats used in this experiment are also common in the PTOA model, consistent with the age stage of young rats [17, 18]. Currently, the treatment strategies for PTOA primarily aim to restore proper kinematic and biomechanical conditions and mitigate the inflammatory response. Nonetheless, research indicates that more than 20% of patients experience unexpected PTOA progression despite these efforts, highlighting the existence of crucial underlying factors beyond inflammatory responses and biomechanics [19, 20]. Anatomically and in terms of cellular origin, cartilage, bone, and muscle are intricately interlinked, constituting what is known as the muscle-bone unit [21]. The muscles are key producers of an array of peptides known as myokines. Most studies suggest that myokines exert autocrine control over muscle metabolism while also engaging in paracrine interactions with distant organs and tissues, such as liver, adipose tissue, bone and brain [22]. In this context, we speculated that manipulation of the muscle-bone unit through the agency of myokines might influence the progression of OA (Fig. 5).

Exercise has been shown to be effective in the management of OA. Our prior investigations have already illustrated that moderate mechanical loading can effectively maintain the integrity of articular cartilage [19]. Our study also showed that treadmill exercise could slow down articular cartilage degeneration and subchondral bone loss. The preventive mechanism of exercise against OA was better elucidated when it was found that skeletal muscle would show the release of myokines under the intervention of exercise [23]. In a recent investigation, it was elucidated that moderate-intensity exercise significantly increased serum irisin levels and bolstered the presence of the anti-inflammatory factor interleukin-10 (IL-10) in elderly women with concurrent sarcopenia and OA. This exercise-induced response effectively repressed the expression of the inflammatory factor tumor necrosis factor-α(TNF-α), consequently leading to a reduction in OA index [24]. Our findings are consistent with the above conclusion that exercise training increases irisin expression and may be involved in alleviating the progression of OA.

Recently, there has been a growing body of studies dedicated to elucidating the protective impact of irisin on OA cartilage tissue. Wang et al. demonstrated that irisin plays a cartilage protective role in the development of OA by promoting mitochondrial biogenesis and maintaining the process of autophagy to resist oxidative stress and catabolism of extracellular matrix under inflammatory response [25]. At the same time, some studies have shown that irisin can reduce joint wear caused by 4 weeks after ACLT, maintain the proportion of hyaline cartilage, promote more complete cartilage structure and lower OARSI score [11]. Irisin is thought to be produced by cleavage of the transmembrane protein FNDC5 [26]. FNDC5, identified as a type I membrane protein, has been recognized as a target gene of PGC-1α in murine models [27]. PGC-1α expressed abundantly in healthy skeletal muscle cells, plays a pivotal role in augmenting mitochondrial biogenesis and enhancing skeletal muscle strength [28]. Indeed, our study showed that rats in the PTOA/Sed group had reduced PGC-1α expression in the quadriceps femoris and severe denervated muscle atrophy, and the expression level of PGC-1α may be closely related to resistance to muscle fiber atrophy. At the same time, some studies have found that PGC-1α is not required for the effect of exercise training on mitochondrial content through biogenesis, because PGC-1α deletion does not prevent exercise-induced mitochondrial biogenesis, suggesting the existence of an independent mechanism other than PGC-1 [29]. While the association between FNDC5 expression, irisin regulation, and exercise in humans has been explored, the mechanisms underlying this connection remain subject to ongoing debate. First of all, the details of FNDC5 cleavage are unknown. Yu et al. demonstrated that a member of the disintegrin and metalloproteinase (ADAM) family, possibly ADAM10, is a candidate for FNDC5 cleavage [30]. Angiotensin II can up-regulate ADAM10, suggesting that angiotensin II may promote FNDC5 cleavage [26]. Secondly, certain studies have found that the expression of FNDC5 in muscle remained unelevated during simulated exercise. Notably, some exercise training regimens that did not observe an upregulation in FNDC5 also failed to demonstrate increased PGC-1α expression, or didn't investigate alterations in PGC-1α levels. This suggests that modifications in FNDC5 expression consequent to exercise could indeed be influenced by the regulatory mechanism of PGC-1α [31]. The discrepancies observed among various studies can likely be attributed to variations in factors such as exercise intensity, mode, sampling time points, and the subjects under investigation. Based on this premise, we observed that treadmill exercise reversed the pronounced down-regulation of PGC-1α and FNDC5 expression levels in muscle tissue of PTOA rats at an exercise intensity set at 15 m/min, 30 min per day duration, 5 days per week and an 8-week intervention. Moreover, exercise stimulated an increase in the concentration of metabolic irisin in the blood of PTOA rats, which was closely correlated with the expression levels of PGC-1α and FNDC5. The outcomes of our study find resonance in the research conducted by Jia et al. as well [14]. Of note, some studies have shown that FNDC5 mRNA expression in skeletal muscle increased after 12 weeks of training in humans, without significant increase in circulating irisin content [13]. However, many studies in mice and humans have shown that endurance exercise can induce irisin [32–34]. Identification and quantification of human irisin in blood by mass spectrometry with stable isotope-enriched control peptides as internal standards have been reported. This precise state-of-the-art method shows that human irisin circulates at a rate of approximately 3.6 ng/ml in sedentary individuals; In individuals undergoing aerobic training, this level increased to approximately 4.3 ng/ml. These data unequivocally demonstrate that human irisin exists, circulates, and is regulated by exercise [34].

Given that our findings have unveiled a potential connection between the PGC-1α/FNDC5/irisin signaling axis and OA, we further embarked on an investigation of the intricate associations between muscle, cartilage, subchondral bone, and irisin levels within the Sham/Sed, PTOA/Sed, and PTOA/TW rat groups. Evidently, the PTOA/Sed group exhibited a decrement in serum irisin metabolism levels. In contrast, the relevant markers exhibited an augmentation in response to treadmill exercise within the PTOA/TW group. Notably, these indicators exhibited a robust negative correlation with both OARSI scores and subchondral bone loss in the PTOA/TW cohort. Our results find resonance in the research conducted by Mao et al. [35], who in their study involving 215 knee OA patients, discovered a negative correlation between serum and joint fluid irisin levels and the severity of human OA, as determined by the Kellgren–Lawrence (KL) grading system. Additionally, Jia et al. demonstrated that treadmill exercise considerably elevated irisin levels in the blood circulation as well as in the synovial fluid of the knee joint, and exercise-induced irisin exerted anti-inflammatory and anti-pyroptotic effects in chondrocytes. This was achieved through the inhibition of the PI3K/Akt/NF-κB signaling pathway, thereby conferring potential for OA treatment [14]. The nexus between irisin and BMD also garners support from various pieces of evidence. In a study conducted by Zhang et al. in 2020, it was shown that irisin levels showed a trend of positive correlation with BMD in older men living in China [36]. To further substantiate the connection between irisin and bone metabolism, Zhu's team carried out a study in 2021 involving FNDC5/irisin knockout mice. Their findings revealed that these knockout mice displayed markedly reduced BMD during both development and adulthood, accompanied by delays in bone development and mineralization. Additionally, the increase in bone thickness typically prompted by wheel running in mice was weakened in irisin knockout individuals [37]. Irisin, by interacting with integrins and activating the Wnt/β-catenin and ERK/MAPK signaling pathways within cells, facilitates bone remodeling [38]. Furthermore, our study unearthed a potent negative correlation between irisin metabolism levels and the expression of Atrogin-1 and MuRF-1 positive tissues, as well as CSA, across the Sham/Sed, PTOA/Sed, and PTOA/TW rat groups. This underpins the notion that irisin's autocrine actions might indeed mitigate muscle atrophy. This premise is substantiated by the research conducted by Guo et al., who observed that aging FNDC5/irisin knockout mice exhibited accelerated loss of muscle mass and strength. Intriguingly, intraperitoneal injection of recombinant irisin protein effectively attenuated age-related sarcopenia and metabolic disorders. Mechanistically, irisin hindered the effects of MuRF1 and Atrogin-1—key players in muscle atrophy—by modulating the ubiquitin–proteasome system [39]. However, the precise downstream pathways of irisin signaling necessitate further investigation. Building upon these collective insights, our hypothesis postulates that exercise may effectively mitigate chondrogenic inflammation through the regulation of the muscle-based PGC-1α/FNDC5/irisin signaling axis. This activation, in turn, could curtail the PI3K/Akt/NF-κB signaling pathway—culminating in a decline in the expression of cartilage matrix-degrading enzymes like MMP-13 and a disintegrin and metalloproteinase with thrombospondin motifs (ADAMTS-5). By intervening at this juncture, exercise could significantly suppress the expression of inflammatory cytokines such as TNF-α and interleukin-1β(IL-1β), both implicated in the apoptotic protease pathway. As a cascading effect, this suppression could help ameliorate the degradation of COL2A1 and bolster the production of glycosaminoglycans within chondrocytes [21, 40, 41]. Concomitantly, exercise exerts a downregulatory influence on the expression of inhibitors tied to the Wnt/β-catenin pathway, which in turn curtails bone loss. This strategic modulation of the pathway serves to invigorate bone remodeling processes [42]. Concurrently, exercise demonstrates its prowess by quelling the expression of MuRF1 and Atrogin-1, pivotal factors in muscle atrophy, by modulating the intricate ubiquitin–proteasome system [36]. This comprehensive orchestration of effects collectively engenders favorable outcomes for individuals grappling with osteoarthritic joints.

Our study remains certain limitations that warrant acknowledgment. To begin with, the size of the rat sample included in our study remains relatively modest. Secondly, this study only revealed the correlation between irisin, PTOA, muscle-bone unit and exercise, and did not deeply explore the regulatory mechanism of irisin with PGC-1α and FNDC5 by regulating target genes. Subsequently, we will verify the relevant mechanism through cell experiments and animal experiments, and provide direct evidence for irisin-mediated signaling pathways by adding irisin inhibitors. In addition, it is crucial to consider the marked biomechanical disparities between human and rat knees, requiring validation through experimental verification in future human cohorts. Finally, the effect of exercise may be due to the short training duration and uniform end time point. Therefore, there should be corresponding improvement programs for different severity of OA animal models. Our future trajectory involves refining our protocol to enhance its applicability to human studies. Subsequent experiments are poised to delve into relevant downstream signaling pathways within the muscle-bone unit, thus offering a more comprehensive perspective on the mechanisms.

In conclusion, exercise significantly ameliorated the severity of PTOA by improving cartilage degeneration, subchondral bone loss and muscle atrophy. In addition, our study suggests that irisin may lead us to a more comprehensive understanding of the beneficial effects of exercise and develop new ideas and therapeutic targets for the treatment of OA in the future.

## Data Availability

Data sharing is not applicable to this article as no datasets were generated or analyzed during the current study.

## Abbreviations

PTOA: Post-traumatic osteoarthritis
OA: Osteoarthritis
SD: Sprague-Dawley
Sed: Sedentary
TW: Treadmill-walking
ACLT: Transection of the anterior cruciate ligament
DMM: Destabilization of the medial meniscus
PGC-1α: Peroxisome proliferator-activated receptor-gamma coactivator-1α
FNDC5: Fibronectin type III structural domain protein 5
OARSI: Osteoarthritis Research Society International
CSA: Cross-sectional area
MuRF-1: Muscle ring-finger protein-1
ACL: Anterior cruciate ligament
BMD: Bone mineral density
PPAR-γ: Peroxisome proliferator-activated receptor-γ
BV/TV: Bone volume/total volume
Tb.N: Trabecular number
Tb.Sp: Trabeculae separation
Tb.Th: Trabecular thickness
EDTA: Ethylenediaminetetraacetic acid
HE: Hematoxylin-eosin
SafO: Safranin O
MTP: Medial tibial plateau
MMP-13: Matrix metalloproteinases -13
COL2A1: Collagen type II alpha 1
ELISA: Enzyme-linked immunosorbent assay
ANOVA: One-way analysis of variance
IL-10: Interleukin-10
ADAM: A disintegrin and metalloproteinase
ADAMTS: A disintegrin and metalloproteinase with thrombospondin motifs
KL: Kellgren–Lawrence
TNF-α: Tumor necrosis factor-α
IL-1β: Interleukin-1β
